# Supplementary material for: Causal Relationship between Aging and Anorexia Nervosa: A White-Matter-Microstructure-Mediated Mendelian Randomization Analysis
Source: Biomedicines. 2024 Aug 16;12(8):1874. doi: 10.3390/biomedicines12081874 (PMC11351342; doi:10.3390/biomedicines12081874)
Supplement: Supplementary file 1 [file biomedicines-12-01874-s001.zip › Supplementary Figure/Figure S8/Leaveoneout plot of MR analysis for TL to ubm-b-1997.pdf]

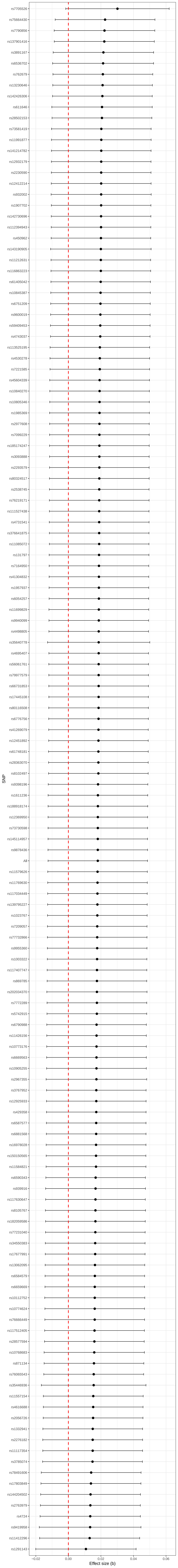
rs7705526
rs75664430
rs7790856
rs137901416
rs3891167
rs6536702
rs762679
rs13230646
rs142426306
rs611646
rs28502153
rs73581419
rs11991877
rs141214782
rs12932179
rs2230590
rs12412214
rs932002
rs1907702
rs142730696
rs112394943
rs450962
rs143190905
rs11212631
rs116863223
rs61405042
rs10845387
rs6751209
rs9600019
rs59409453
rs4743037
rs113525195
rs4530278
rs7221585
rs45604339
rs10840270
rs10805346
rs1985369
rs2977608
rs7099229
rs185174247
rs3093888
rs2293579
rs80324517
rs2538745
rs76219171
rs111527438
rs4731541
rs376641875
rs11085072
rs131797
rs7164950
rs41304832
rs1957937
rs6054257
rs11699829
rs9940099
rs4498805
rs35640778
rs4695407
rs56061761
rs79977579
rs66731853
rs17445108
rs80116508
rs6776756
rs41269079
rs12451892
rs61748181
rs28363070
rs8102497
rs9398196
rs1611236
rs188918174
rs12369950
rs73730598
rs145114957
rs9878436
All
rs11579626
rs11769630
rs117034449
rs139795227
rs1023767
rs7209057
rs77732866
rs9955360
rs1003322
rs117407747
rs869785
rs202034370
rs7772289
rs5742915
rs6790988
rs11426156
rs10773176
rs6669563
rs10905255
rs2967355
rs3767952
rs12925933
rs429358
rs6587577
rs6881568
rs16978028
rs150150565
rs11584821
rs6590343
rs939916
rs117630647
rs8105767
rs182059586
rs77231040
rs34550383
rs17677991
rs13062095
rs6584579
rs6659669
rs10112752
rs10774624
rs76666449
rs117512405
rs28577594
rs10768683
rs871134
rs76065543
rs35446936
rs11557154
rs4616688
rs2056726
rs1332941
rs2276182
rs11117354
rs3785074
rs78491606
rs17803849
rs144204502
rs2763979
rs4724
rs9419958
rs11412296
rs1291143
SNP
−0.02
0.00
0.02
0.04
0.06
Effect size (b)
